# Long-term mortality in patients with a prolonged postoperative intensive care unit (PICU) stay

**DOI:** 10.1186/2197-425X-3-S1-A740

**Published:** 2015-10-01

**Authors:** M Guerrero Díez, Á Callejo Martin, C Camús Sánchez, A Calderón, M Levstek, A Hermira Anchuelo, M González Serrano, E López López

**Affiliations:** Hospital Universitario 12 Octubre, Anaesthesiology and Intensive Care, Madrid, Spain

## Introduction

Non-cardiac surgical patients undergoing major surgery are progressively older and with multiple associated comorbidity. A worse baseline status and complicated postoperative course is usually assumed in patients who die in the postoperative intensive care unit (PICU). This study evaluates mortality in patients with prolonged ICU stay and try to identify differences in subjects characteristics and to determine risk factors for mortality among those dying in ICU.

## Methods

A total of 429 patients who underwent major surgery between January 2012 and December 2013, with an ICU stay of 2 days or more, were included in the study. We compared demographic characteristics (age, sex and ASA classification), type of surgery (emergent or not and reinterventions) and postoperative course variables (presence of vasoactive drugs, days of mechanical ventilation (MV) and immediate postoperative albumin and bilirrubin levels) between patients who died or not in PICU. Results were expressed as mean ± SD for continuous variables or as a percentage of the group from which they derive for qualitative ones. A t-Student test was performed to assess whether the differences in means values of qualitative variables were statistically significant. Distribution of categorical variables was compared by Fisher´s exact test. Odds ratios (OR) were calculated to identify risk factors for mortality. A p-value < 0.05 was considered statistically significant for all the analysis.

## Results

50 (11.65%) out of 429 patients died in our unit after staying 2 or more days. Statistically significant difference in age, use of vasoactive drugs and time of MV was observed between the 2 groups. In the univariate analysis, despite a wide confidence interval (CI), a 4 and 2.5 times increased risk of death was observed due to vasoactive drugs and prolonged mechanical ventilation, respectively. An increasing risk for advancing age was suggested but was not statistically significant. Results are summarized in Table 1.

**Table 1:** Table 1

		DEATHS	SURVIVALS	p-value	OR	CI
**SEX**	Male Female	27 (54%) 23 (46%)	233 (61,48%) 146 (38,52%)	0,0722		
**AGE (mean ± SD)**		71,92 ± 14,20	67,04 (15,46)	0,0273	1,55	0,83 - 2,87
**ASA**	I II III IV	0 (0%) 10 (20%) 30 (60%) 10 (20%)	7 (1,85%) 110 (29,02%) 188 (49,6%) 74 (19,53%)	0,1826		
**EMERGENCY**	Yes No	27 (54%) 23 (46%)	203 (53,56%) 176 (46,44%)	0,1196		
**REINTERVENTION**	Yes No	18 (36%) 32 (64%)	117 (39,87%) 262 (69,13%)	0,0960		
**VASOACTIVE DRUGS**	Yes No	43 (86%) 7 (14%)	221 (58,31%) 158 (41,69%)	< 0.01	4,39	1,93 - 10,02
**DURATION of MV (mean ± SD)**		10,64 (14,57)	3,14 (7,39)	< 0.01	2,44	1,28-4,65
**ALBUMIN (mean ± SD)**		2,57 (0,87)	2,68 (0,74)	0,3967		
**BILIRUBIN (mean ± SD)**		1,09 (0,90)	1,15 (1,23)	0,6613		

## Conclusions

Patients with a long stay who died in PICU were older and showed worse postoperative course. Use of vasoactive drugs and prolonged mechanical ventilation were identified as significant risk factors. Multivariate analysis should be performed to assess them as independent risk factors. Although these findings are consequent with literature, larger trials will be necessary to study more variables and estimate predictors of mortality in PICU.
